# Emerging roles of circular RNAs in tumorigenesis, progression, and treatment of gastric cancer

**DOI:** 10.1186/s12967-024-05001-4

**Published:** 2024-02-27

**Authors:** Qiang Ma, Feifei Yang, Bin Xiao, Xiaolan Guo

**Affiliations:** 1 Department of Clinical Laboratory, Affiliated Hospital of North Sichuan Medical College, Nanchong, 637000 People’s Republic of China; 2 Translational Medicine Research Center & School of Laboratory Medicine, North Sichuan Medical College, Nanchong, 637000 People’s Republic of China; 3 College of Pharmacy, Chongqing Medical University, Chongqing, 400016 People’s Republic of China

**Keywords:** circRNA, Gastric cancer, miRNA sponge, RNA binding protein, Translational template, Biotechnological drugs

## Abstract

With an estimated one million new cases reported annually, gastric cancer (GC) ranks as the fifth most diagnosed malignancy worldwide. The early detection of GC remains a major challenge, and the prognosis worsens either when patients develop resistance to chemotherapy or radiotherapy or when the cancer metastasizes. The precise pathogenesis underlying GC is not well understood, which further complicates its treatment. Circular RNAs (circRNAs), a recently discovered class of noncoding RNAs that originate from parental genes through “back-splicing”, have been shown to play a key role in various biological processes in both eukaryotes and prokaryotes. CircRNAs have been linked to cardiovascular diseases, diabetes, hypertension, Alzheimer's disease, and the occurrence and progression of tumors. Prior studies have established that circRNAs play a crucial role in GC, impacting tumorigenesis, diagnosis, progression, and therapy resistance. This review aims to summarize how circRNAs contribute to GC tumorigenesis and progression, examine their roles in the development of drug resistance, discuss their potential as biotechnological drugs, and summarize their response to therapeutic drugs and microorganism in GC.

## Introduction

Gastric cancer (GC) is the fifth most incident cancer and the third leading cause of tumor-related mortality worldwide. Its highest incidence and mortality rates have been recorded in East Asia, Eastern Europe, and South America [1, 2]. Risk factors for GC include *Helicobacter pylori* infection, low socio­economic status, advanced age, cigarette smoking, alcohol consumption, genetic predisposition, previous gastric surgery, pernicious anemia, and living in a high-risk population [2]. The prognosis of patients with GC is closely related to classification of the disease. The Borrmann system, which including types I–IV, is the most commonly used macroscopic classification system for GC [3]. It is simple, practical, and can indicate the biological behavior of GC. Histologically, GC can be classified according to Lauren’s criteria [4] and the World Health Organization system [5]. Lauren’s criteria, which is widely used clinically, classifies GC into three types: intestinal, diffuse, and mixed. The World Health Organization classification system of gastric adenocarcinoma is not widely used because of its complexity in distinguishing between many subtypes, besides the fact that some of the subtypes are very rare.

These GC classification systems have enabled significant advancements in diagnostic and therapeutic technologies in recent years, but patient prognosis remains poor, primarily because of the high recurrence rate and low 5 year overall survival rate of GC [6]. Currently, chemotherapy, radiotherapy, and immunotherapy are the common treatments for GC, but they elicit side effects and produce unsatisfactory therapeutic outcomes. Elucidating the molecular mechanisms that govern the tumorigenesis and progression of GC can crucially guide clinical practice, help predict prognosis, and estimate the response to treatment.

The human genome contains about 30,000 coding genes, while approximately 98.5% of the transcriptome consists of noncoding RNAs, such as micro RNAs (miRNAs), long noncoding RNAs, circular RNAs (circRNAs), piwi-interacting RNAs, and small nucleolar RNAs, which regulate gene expression through various mechanisms [7–9]. CircRNAs are noncoding RNAs with a covalently closed loop structure that lacks a 5′ cap and a 3ʹ polyadenylate tail [10]. In 1976, Kolakofsky D observed ring-shaped RNA molecules within the Sendai virus for the first time [11], while circRNA molecules were reported in the cytoplasm of eukaryotic cells for the first time by Hsu et al. [12]. However, the structure, biogenesis, and biological functions of circRNAs could not be elucidated in the subsequent decades due to technological limitations. With the advent of sequencing technology and bioinformatic tools, circRNAs have now been discovered in various species, including humans, and have been found to play crucial roles in individual development and in the pathogenesis of various diseases.

RNA splicing is a crucial step in the expression of eukaryotic genes. During mRNA maturation, introns are removed from pre-mRNA molecules, and exons are covalently bonded to each other to form mature mRNAs. In contrast, circRNAs are formed by “back splicing”, which often involves exon skipping [13], cis/trans element-mediated circularization [14], lariat-mediated circularization, intron pairing-driven circularization [10], or splicing of pre-tRNAs [15]. CircRNAs can be classified into four types based on their composition of introns or exons: exon-derived circRNAs, intron-derived circRNAs, circRNAs containing both exons and introns, and tRNA-derived circRNAs [16]. In recent years, numerous circRNAs, such as circ-SHPRH, circFNDC3B, circGRFA1, and circCCDC66, were shown to be dysregulated in various tumors and to be linked to tumorigenesis, tumor progression, and treatment outcome [17–20]. Moreover, some exosome-encapsulated circRNAs can be used as diagnostic biomarkers for tumors and were involved in mediating drug resistance to anti-tumor therapy [21, 22].

This article reviews the various roles played by circRNAs in different aspects of GC, including tumorigenesis, progression, diagnosis, and therapeutic resistance. Further, we investigate the clinical potential of circRNAs as prognostic biomarkers and drugs for GC. Finally, we examine the importance of circRNAs in the context of GC patients who have been treated with specific drugs or previously infected with *H. pylori*.

This review is particularly innovative for three reasons. First, we have meticulously reviewed the most recent literature to conceptualize the structure of this review around the molecular workings of circRNAs. Second, the discussion on the utility of circRNAs as biotechnological therapeutics in treating gastric cancer marks a significant contribution. Finally, the examination of circRNAs modulation by pharmaceuticals or microorganisms in GC sets this review apart from existing literature on the subject.

## CircRNAs contribute to tumorigenesis and progression of GC through diverse mechanisms

High-throughput technology has enabled the detection of circRNAs in GC tissues and cell lines. Wang et al. [23] identified 75,201 circRNAs in GC tissues and cell lines using next-generation sequencing. Bu et al. [24] identified 28,137 circRNAs in five pairs of GC and adjacent tissues using RNA sequencing. Therefore, circRNAs are no longer considered to be “dark matter” in humans but rather play important roles in physiological development as well as various diseases, including cancers.

CircRNAs can function as miRNA “sponges” or interact with RNA-binding proteins (RBPs) and other proteins. Additionally, circRNAs can serve as templates for translation, regulate alternative splicing, and influence mRNA transcription in tumors [14]. In this section, we will summarize the diverse roles of circRNAs in tumorigenesis and progression of GC.

### CircRNAs function as miRNA “sponges” in GC

CircRNAs can fulfil oncogenic roles in GC by “sponging” miRNAs, thereby indirectly regulating expression of downstream target genes. Hsa_circ_0000267, a circRNA derived from *FAM53B* that was significantly overexpressed in GC tissues and cell lines, adsorbs miR-503-5p, which upregulates *HMGA2* and promotes the onset of GC [25]. Li et al. [26] found that circBGN was significantly upregulated in GC tissues and cells, promoting proliferation and invasion of GC both in vitro and in vivo. A subsequent study revealed that circBGN directly sequestered miR-149-5p to prevent it from binding to interleukin-6 mRNA, ultimately activating the interleukin-6/signal transducer and activator of transcription 3 signaling pathway and playing an oncogenic role in GC. Moreover, downregulation of methyltransferase-like protein 14 served as a prognostic factor indicating poor survival in GC patients. Mechanistically, methyltransferase-like protein 14 was found to inhibit circORC5 by mediating its *N*^*6*^-methyladenosine modification. Given that circORC5 sponges miR-30c-2-3p, its inhibition ultimately suppressed GC progression via the miR-30c-2-3p/AKT1 substrate 1 axis [27].

In addition to their oncogenic effects, some circRNAs have been identified as tumor suppressors in GC. CircRAB31, which originates from exons 2–5 of *RAB31*, was significantly downregulated in GC tissues and cell lines. By adsorbing miR-885-5p, circRAB31 can modulate the phosphatase and tensin homolog/phosphoinositide 3-kinase (PI3K)/AKT signaling pathway, eventually mitigating proliferation and metastasis of GC cells [28]. Similarly, circST3GAL6 was found to be significantly downregulated in GC tissues. CircST3GAL6 can adsorb miR-300 to upregulate *FOXP2* and downregulate MET/AKT/mammalian target of rapamycin signaling, which results in inhibition of both GC proliferation and metastasis [29]. Thus, the circRNA–miRNA axis plays a crucial regulatory role in initiation and progression of GC.

### CircRNAs interact with RBPs or other functional proteins in GC

**Fig. 1:**
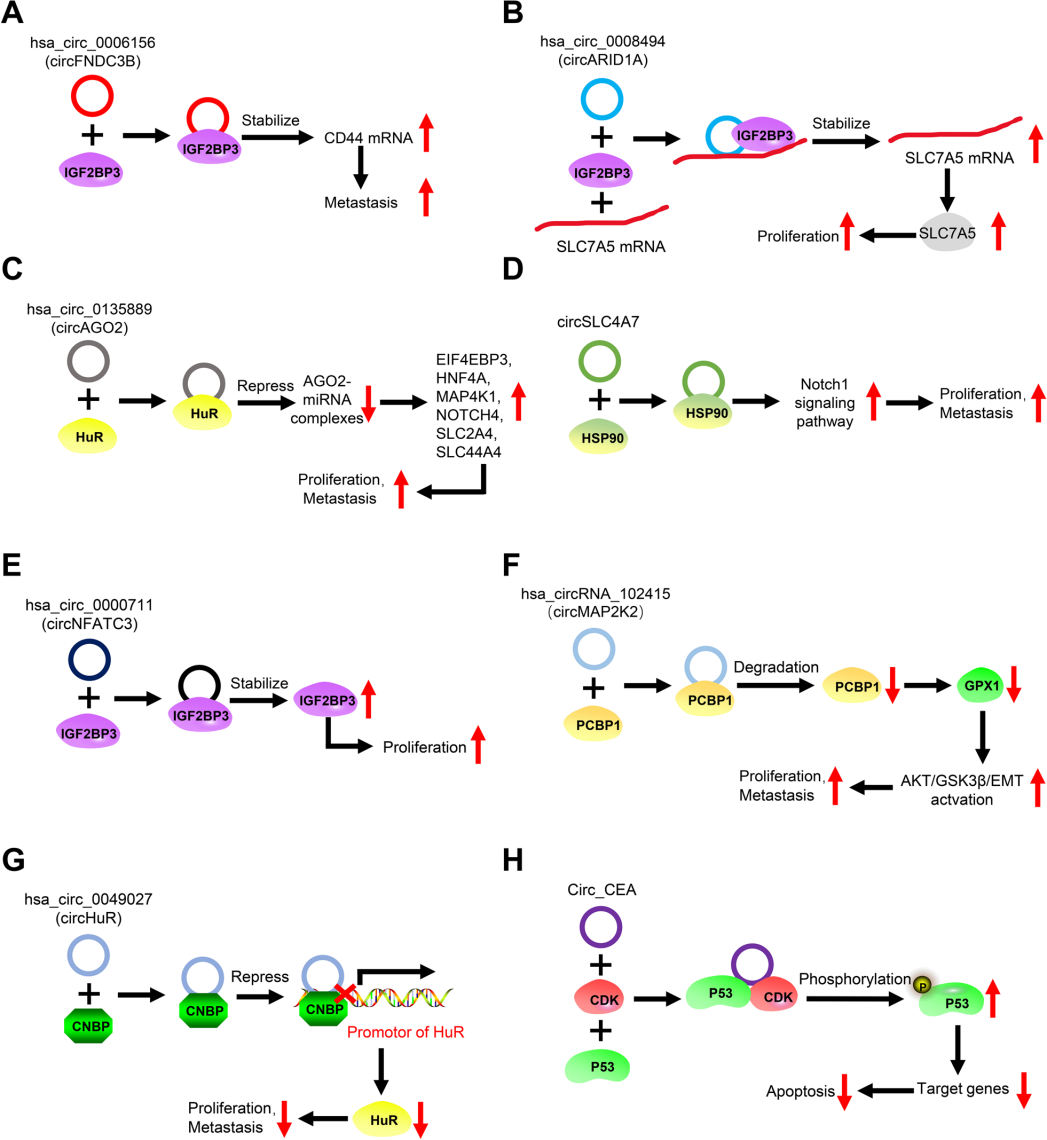
The roles of circRNAs involving interaction with RBPs or other proteins in GC. **A**. The circular RNA hsa_circ_0006156 (circFNDC3B) interacts with IGF2BP3 in GC cells, leading to increased stability of CD44 mRNA, which ultimately promotes the migration and invasion of GC. **B**. Hsa_circ_0008494 (circARID1A) interacts with IGF2BP3 in GC cells to form an hsa_circ_0008494–IGF2BP3–SLC7A5 ternary complex that enhances the expression of SLC7A5 and ultimately, promotes GC proliferation. **C**. Hsa_circ_0135889 (circAGO2) interacts with HuR, inhibiting the function of the AGO2–miRNA complex, which leads to the upregulation of downstream target genes and promotes proliferation and metastasis of GC. **D**. CircSLC4A7 interacts with HSP90 and activates the Notch1 signaling pathway to promote the proliferation and metastasis of GC. **E**. Hsa_circ_0000711 (circNFATC3) binds to and prevents the degradation of IGF2BP3 to promote the proliferation of GC. **F**. Hsa_circRNA_102415 interacts with and promotes the degradation of PCBP1, which inhibits GPX1 expression and finally promotes the proliferation and metastasis of GC cells by activating AKT/GSK3β/EMT signaling. **G**. Hsa_circ_0049027 (circHuR) interacts with the transcription factor CNBP to repress HuR expression, which inhibits the proliferation and metastasis of GC. **H**. Circ_CEA serves as a “scaffold” facilitating the interaction between CDK and P53, which promotes the phosphorylation of P53 protein and the downregulation of downstream genes. Eventually, the apoptosis of GC cells is inhibited

CircRNAs can bind to RBPs or other functional proteins to regulate the expression of target genes and ultimately, control the growth and metastasis of GC (Fig. 1A–D). Hsa_circ_0006156, a circRNA derived from *FNDC3B*, can bind to insulin-like growth factor 2 mRNA-binding protein 3 (IGF2BP3) in GC cells to enhance IGF2BP3-mediated stability of CD44 mRNA, eventually promoting GC metastasis[30]. Moreover, circARID1A was also reported to be significantly upregulated in GC tissues and was positively correlated with tumor length, tumor volume, and TNM stage [31]. Mechanistically, circARID1A forms an RNA–protein ternary complex with IGF2BP3 and *SLC7A5* mRNA, which stabilizes *SLC7A5* mRNA and promotes cell proliferation in GC [31]. Furthermore, being the core component of the miRNA-induced silencing complex, Argonaute 2 (AGO2) is indispensable for miRNA function. According to Chen et al. [32], hsa_circ_0135889, derived from *AGO2*, is highly expressed in various tumors, including GC, which it can bind to and activate the RBP human antigen R (HuR), thereby restraining the function of the AGO2–miRNA complex. Finally, hsa_circ_0135889 indirectly upregulates downstream target genes to exert its oncogenic role. In addition, circSLC4A7 was found to be one of the most upregulated circRNAs in GC stem cells, wherein it localized to the nucleus. Mechanistically, circSLC4A7 activates Notch1 signaling by binding to heat shock protein 90, thus promoting cancer stem cell-like properties and inducing cell proliferation, migration, and invasion [33].

Besides regulating the expression of target genes, some circRNAs were also found to regulate the stability of RBPs (Fig. 1E–F). For example, circNFATC3, which is upregulated in GC and positively correlated with tumor progression, binds to and enhances the stability of IGF2BP3 by reducing its ubiquitination and degradation via the proteasome pathway. Thus, IGF2BP3 overexpression is partially stimulated by the upregulation of circNFATC3, which ultimately facilitates the proliferation of GC cells [34]. Moreover, circMAP2K2, which is upregulated in GC, regulates the poly(rC)-binding protein 1/glutathione peroxidase 1 axis through proteasome-mediated degradation, and activates the AKT/glycogen synthase kinase-3β/epithelial-to-mesenchymal transition signaling pathway to enhance GC cell proliferation and metastasis [35].

Besides RBPs, circRNAs do bind to other functional protein. They can influence GC progression by regulating the expression of their parent genes through transcription factors (Fig. 1G). Hsa_circ_0049027, which is derived from *HuR* and is significantly downregulated in GC tissues and cell lines. Functionally, hsa_circ_0049027 binds to the transcription factor CCHC-type zinc finger nucleic acid-binding protein, thereby prevents it from binding to the *HuR* promoter and inhibits *HuR* expression, ultimately hinders the progression of GC [36].

Interestingly, circRNAs have also been shown to significantly influence protein phosphorylation (Fig. 1H). Circ_CEA is significantly upregulated in GC tissues and cell lines, wherein it serves as a scaffold to facilitate the interaction between p53 and cyclin-dependent kinase 1, which phosphorylates p53 at Ser315, decreases its retention in the nucleus, and suppresses its activity. Consequently, p53 target genes associated with apoptosis are downregulated and GC cell apoptosis is inhibited [37].

**Fig. 2:**
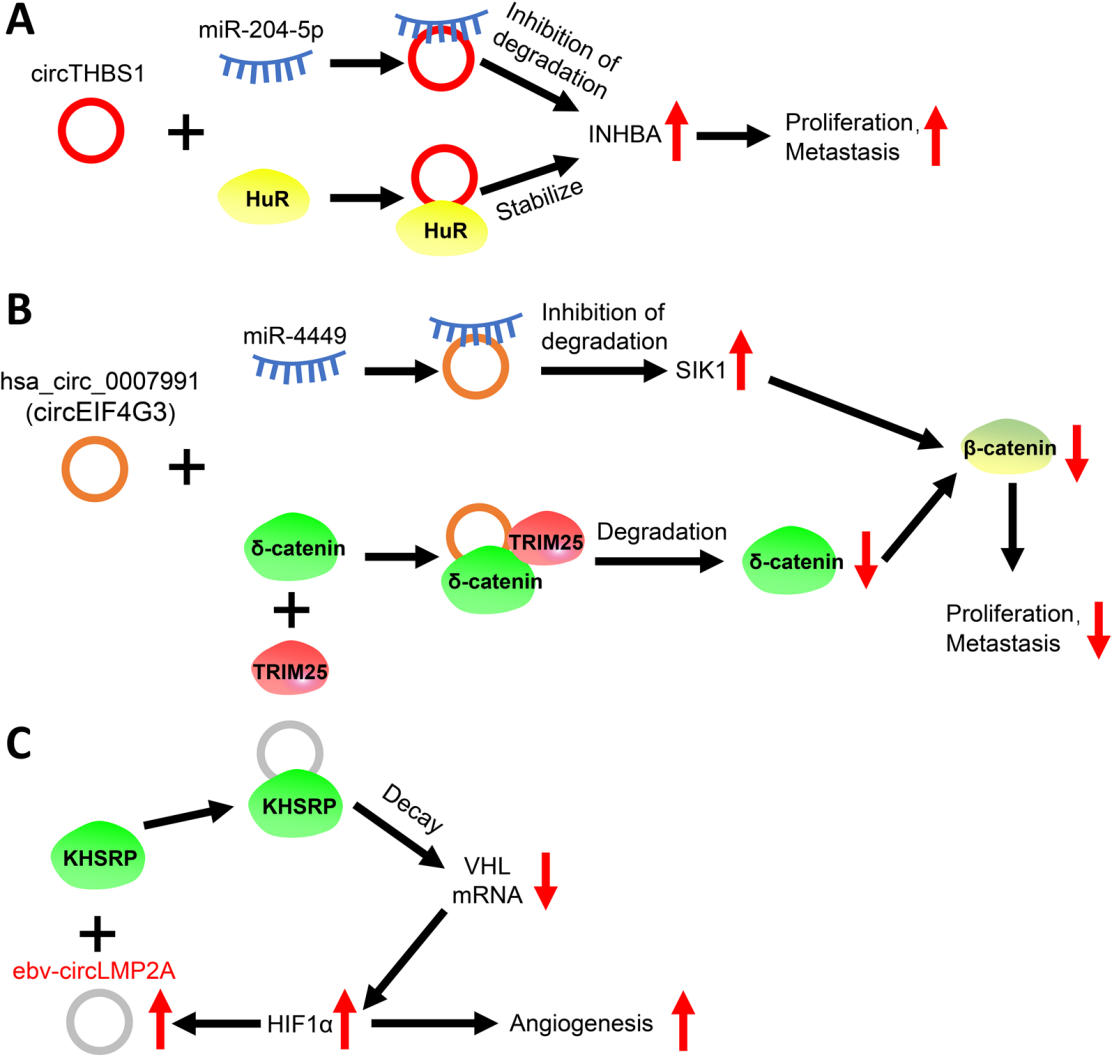
The dual roles of circRNAs in regulating GC tumorigenesis or progression and the role of virus-derived circRNAs in GC progression. **A**. CircTHBS1 promotes GC proliferation and metastasis by upregulating INHBA by sponging miR-204-5p and interacting with HuR. **B**. CircEIF4G3 interacts with and enhances the degradation of δ-catenin and upregulating SIK1 through miR-4449 sponging, which ultimately promotes GC proliferation and metastasis by downregulating β-catenin. **C**. The HIF1α-induced EB virus-derived ebv-circLMP2A interacts with KHSRP protein to enhance the decay of VHL mRNA. This mechanism, along with the enhanced expression of HIF1α, forms a regulated loop that ultimately promotes angiogenesis in GC

Some circRNAs can simultaneously sponge miRNAs and interact with RBPs (Fig. 2A–B). CircTHBS1, known to be significantly highly expressed in GC and associated with poor prognosis, sponges miR-204-5p to upregulate *INHBA* and interactes with HuR to enhance the mRNA stability of *INHBA*, ultimately supporting GC cell proliferation and migration [38]. Zang et al. discovered that circEIF4G3 is downregulated in GC and associated with poor overall survival. CircEIF4G3 sponges miR-4449 to upregulate salt-inducible kinase-1. Additionally, it binds to δ-catenin to promote the ubiquitin-mediated degradation of δ-catenin via tripartite motif-containing protein 25. In short, circEIF4G3 downregulated δ-catenin protein by enhancing TRIM25-mediated ubiquitin degradation and functions as a miRNA “sponge” to modulate miR-4449/SIK1 axis, which synergistically leads to the inactivation of β-catenin signaling and the inhibition of GC progression [39].

Interestingly, virus-derived circRNAs have also been linked with GC progression (Fig. 2C). CircLMP2A, encoded within the Epstein–Barr virus genome, is expressed in Epstein–Barr virus-associated GC [40]. Induced by hypoxia-inducible factor-1α (HIF-1α), circLMP2A interactes with KH-type splicing regulatory protein and facilitates the decay of *VHL* mRNA, leading to the accumulation of HIF-1α under hypoxic conditions, which eventually promotes angiogenesis in GC tissues [40].

Taken together, these findings highlight both the oncogenic and tumor-suppressing activities of circRNAs during GC tumorigenesis and progression, which are fulfilled through interactions with RBPs as well as non-RBPs.

### CircRNAs can be used as a template for translation in GC

**Fig. 3:**
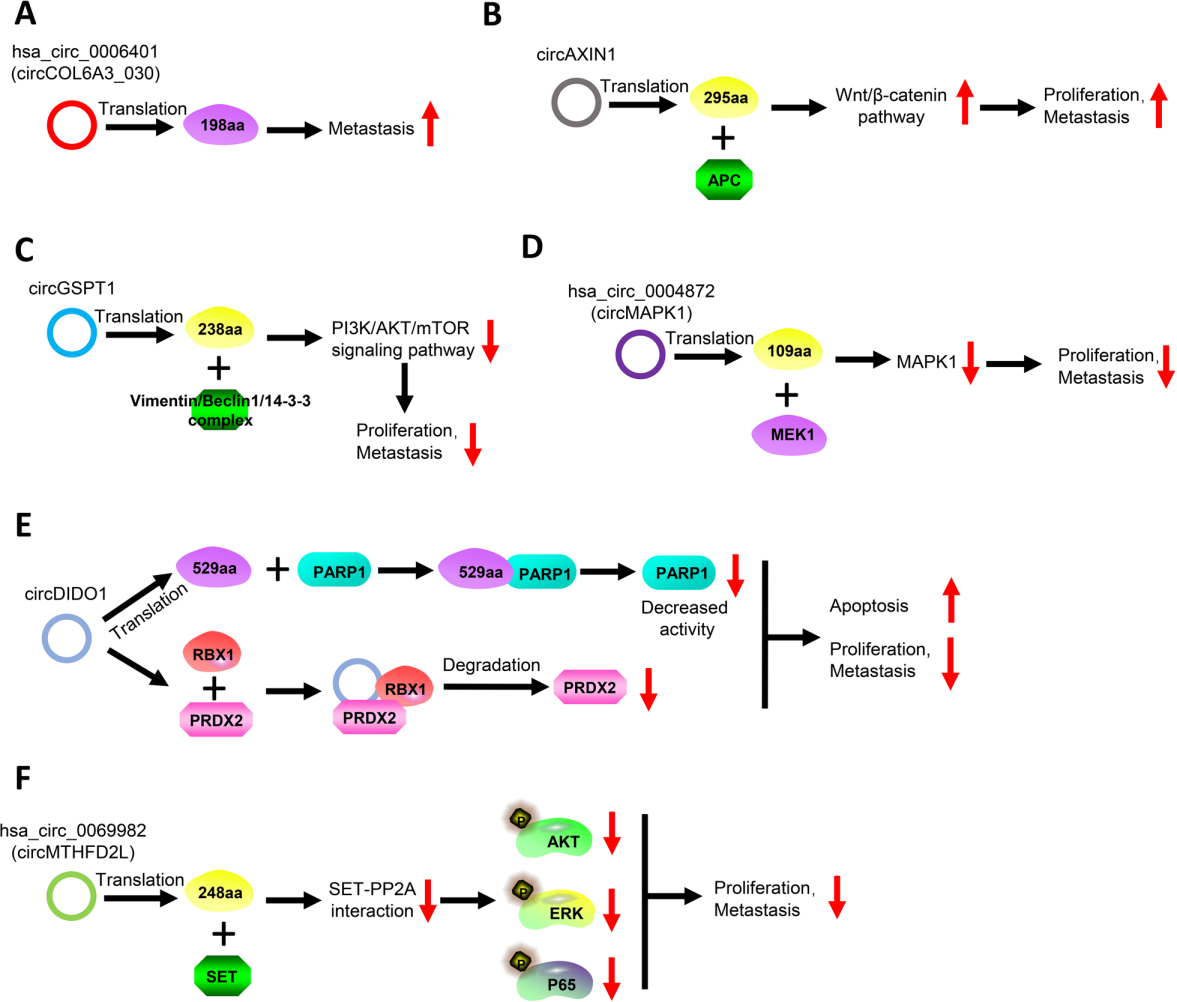
CircRNAs work as translational templates to contribute to the proliferation and progression of GC. **A**. CircCOL6A3_030 encodes a 198-amino-acid peptide that promotes the metastasis of GC through currently unknown mechanisms. **B**. CircAXIN1 produces a 295-amino-acid peptide that interacts with APC to activate the Wnt/β-catenin pathway, ultimately promoting the proliferation of GC. **C**. CircGSPT1 gets translated into a 283-amino-acids-long peptide that interacts with the vimentin/beclin1/14-3-3 complex, inhibiting the PI3K/AKT/mTOR signaling pathway and suppressing the proliferation and metastasis of GC. **D**. Hsa_circ_0004872 (circMAPK1) encodes a 109-amino-acid peptide that interacts with MEK1 to inhibit MAPK1 expression, thereby inhibiting the proliferation and metastasis of GC. **E**. A 529-amino-acid peptide is translated from circDIDO1, which interacts with PARP1 and decrease PARP1 activity. Moreover, circDIDO1 bind to both RBX1 and PRDX2 to promote the degradation of PRDX2. This process finally inhibits the proliferation and metastasis and promotes the apoptosis of GC. **F**. Hsa_circ_0069982 (circMTHFD2L) encodes a 248-amino-acid peptide that disrupts the interaction between SET and PP2A, which attenuates the phosphorylation of AKT, ERK, and P65 and thus, inhibits the proliferation and metastasis of GC

Some circRNAs with translational potential have been found to contribute to the carcinogenesis and development of GC (Fig. 3A–C). CircCOL6A3_030 is significantly higher in metastatic GC tissues than in non-metastatic GC tissues, which translated into a peptide of 198 amino acids and supports the metastasis of GC cells [41]. Similarly, circAXIN1 is significantly upregulated in GC and can be detected in the form of a novel 295-amino-acid-long protein. AXIN1-295aa activates Wnt/β-catenin signaling by competing with axis inhibition protein 1 and binding to adenomatous polyposis coli, eventually promoting the proliferation and metastasis of GC cells by elevating the expression of Wnt-response genes (Cyclin D1, c-Myc, c-Jun, etc.) [42]. Similarly, circGSPT1, which encodes a 238-amino acid-long protein, is downregulated in GC and suppresses its proliferation, migration, and invasion. GSPT1-238aa interacted with the vimentin/Beclin1/14-3-3 complex and modulates autophagy via the PI3K/AKT/mammalian target of rapamycin signaling pathway in GC cells [43].

CircRNA-encoded peptides can also regulate the function of proteins encoded by their parent genes (Fig. 3D–E). MAPK1-109aa, encoded by circMAPK1 (hsa_circ_0004872), can inhibit the phosphorylation of mitogen-activated protein kinase 1 (MAPK1) by competitively binding to mitogen-activated protein kinase kinase 1, thereby suppressing the activation of MAPK1 and its downstream factors in MAPK pathway, finally attenuating the proliferation and metastasis of GC cells [44]. CircDIDO1, which encodes a novel protein of 529 amino acids, is significantly downregulated in GC tissues. On the one hand, circDIDO1-529aa directly binds to poly (ADP-ribose) polymerase 1 and inhibits its activity. On the other hand, circDIDO1 mitigates the proliferation and metastasis of GC cells by binding to peroxiredoxin-2 and promoting its degradation through E3 ubiquitin-protein ligase RBX1 mediated ubiquitination [45].

Another study showed that circRNA-encoded peptides can regulate the phosphorylation of multiple downstream proteins (Fig. 3F). For instance, circMTHFD2L (hsa_circ_0069982), which encodes a peptide named CM-248aa, is downregulated in GC. Mechanistically, CM-248aa competitively targets the acidic domain of SET nuclear oncogene (SET), endogenously inhibiting the SET–protein phosphatase 2A interaction to promote the dephosphorylation of AKT, extracellular signal-regulated kinase, and p65, which ultimately inhibits the proliferation and metastasis of GC cells [46].

In summary, protein-coding circRNAs commonly exist in GC cells, and the peptides they encode can interact with other proteins to regulate GC tumorigenesis and progression.

### CircRNAs regulate the alternative splicing and transcription of mRNAs in GC

**Fig. 4:**
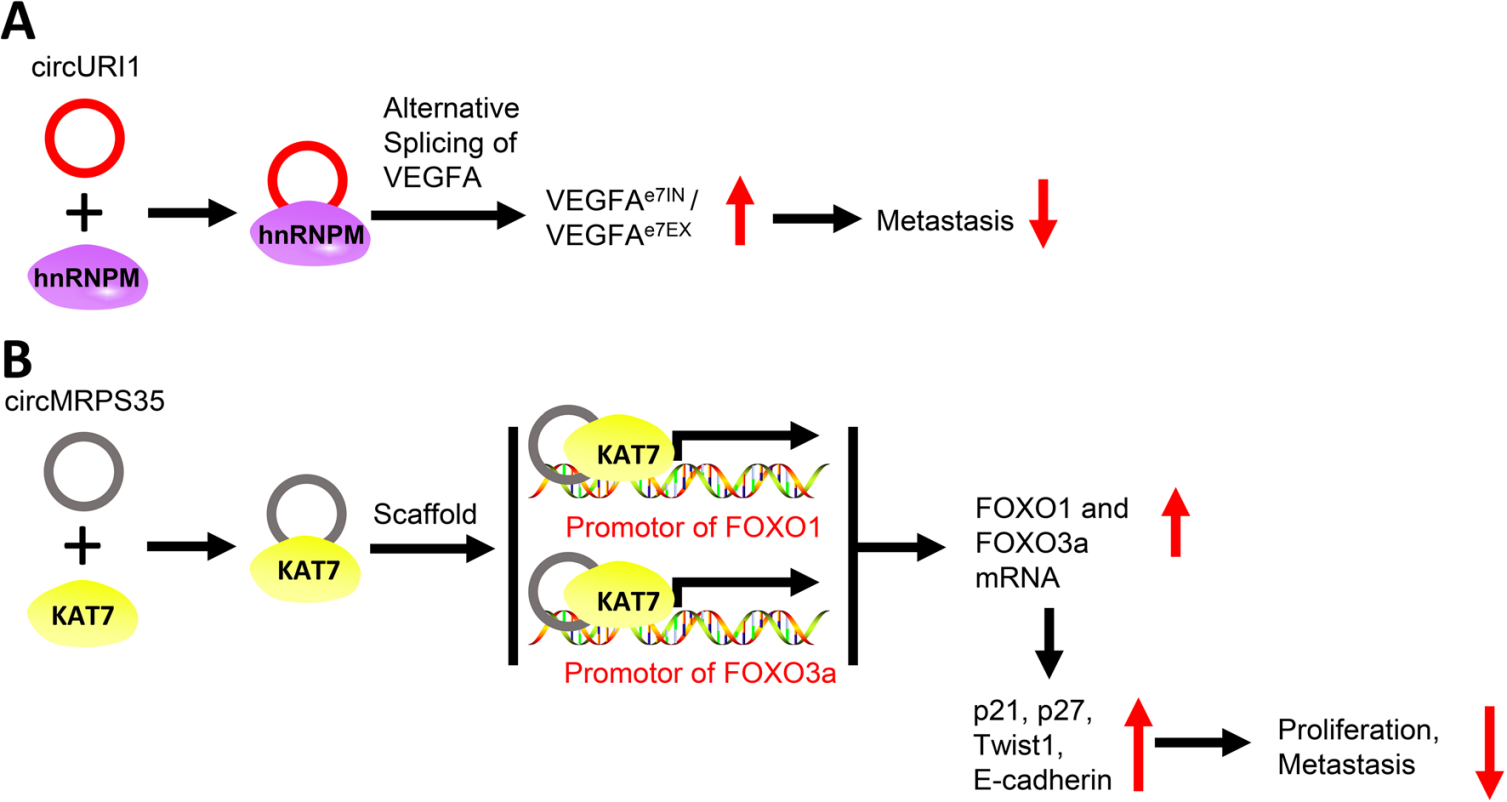
CircRNAs regulate alternative splicing and mRNA transcription in GC. **A**. CircURI1 binds to hnRNPM and regulates alternative splicing of the *VEGFA* gene to elevate the VEGFA^e7IN^/VEGFA^e7EX^ ratio, ultimately inhibiting GC metastasis. **B**. CircMRPS35 interacts with KAT7 and recruits it to the promoter regions of *FOXO1* and *FOXO3a* to activate their transcription, which eventually inhibits the proliferation and metastasis of GC

CircRNAs can also influence the progression of GC by regulating alternative splicing or participating in epigenetic modifications (Fig. 4). For example, circURI1 was found to be expressed significantly higher in GC tissues than in adjacent tissues, and knocking it down enhanced the metastasis of GC cells. CircURI1 binds to heterogeneous nuclear ribonucleoprotein M, regulates the alternative splicing of *VEGFA*, and elevates the VEGFA^e7IN^/VEGFA^e7EX^ ratio to eventually inhibit GC cell metastasis [47]. Conversely, the expression of circMRPS35 was significantly reduced in GC tissues and was associated with poor prognosis. CircMRPS35 activates the transcription of *FOXO1* and *FOXO3a* by acting as a “scaffold” to recruit the histone acetyltransferase KAT7 to the promoter regions of these genes through directly bound to *FOXO1/3a* promoter regions. This leads to the upregulation of downstream target genes, such as p21, p27, Twist1, and E-cadherin, and ultimately inhibits the proliferation and invasion of GC cells [48].

In summary, circRNAs contribute to the carcinogenesis and progression of GC via different mechanisms, and blocking or activating the corresponding signaling pathways represents a new approach for the treatment of GC.

## Circulating circRNAs hold potential as diagnostic markers for GC

The invasiveness of digestive endoscopy makes the diagnosis and treatment of digestive diseases challenging. GC is often diagnosed only at an advanced stage, leading to high mortality rates globally. Traditional tumor markers, such as carbohydrate antigen 19–9 (CA 19–9), carcinoembryonic antigen (CEA), CA 72–4, and pepsinogen I/II, have poor diagnostic specificity for GC. Interestingly, recent studies have indicated that some circRNAs carry diagnostic potential for GC. The expression of hsa_circ_0065149, hsa_circ_0001789, hsa_circ_0086720, and hsa_circ_0005654 was found to be significantly altered in GC tissues and plasma samples [49–52]. Hsa_circ_0065149 was downregulated in the tumor tissues and plasma exosomes of patients with early-stage GC, with an area under the curve (AUC) of 0.640 for early GC diagnosis [49]. Hsa_circ_0001789 was similarly downregulated in GC tissues and plasma samples, with an AUC of 0.82 for diagnosis [50]. Hsa_circ_0086720 was downregulated in GC cells and actively secreted by them, whereas it was stable in circulating plasma [51]. The AUC of plasma hsa_circ_0086720 is more diagnostically valuable for early GC than the AUC for hsa_circ_0086720 from gastric cancer tissue [51]. The expression of hsa_circ_0005654 was significantly inhibited in early GC tissues, with an AUC of 0.924 for early diagnosis [52]. The diagnostic and prognostic value of serum circHAS2, circLPAR1, and exosomal hsa_circ_0079439 has also been demonstrated in GC [53–55]. These findings suggest that circRNAs can serve as promising biomarkers for the early diagnosis of GC. Future studies must validate these findings and investigate the mechanisms underlying circRNA dysregulation in GC.

A combination of CircRNAs with current GC-related markers has exhibited improved the diagnostic efficiency of GC. The combined detection of CEA, CA 19–9, and hsa_circ_0015286, a circRNA upregulated in GC tissues, plasma, and cancer cells, yielded the highest AUC of 0.843 for the early diagnosis of GC, compared with the AUC values for the individual markers [56]. Moreover, the expression of hsa_circ_0006848 decreases significantly in early GC tissues and plasma but increases significantly after surgery. The AUC of plasma hsa_circ_0006848 for the early diagnosis of GC reached 0.733, while it went up to 0.825 when the circRNA was combined with CEA, CA 19–9, and CA 72–4 [57]. Furthermore, the combination of multiple circRNAs also showed high diagnostic efficacy. Roy et al. identified a diagnostic panel for GC comprising eight serum circRNAs. The AUC of the panel was 0.83, implying that it could efficiently distinguish patients with GC from healthy individuals [58]. These studies underscore the potential of circRNAs in the early diagnosis of GC.

Studies have also explored the diagnostic efficacy of circRNAs for advanced GC. Hsa_circ_0000467 is significantly upregulated in GC but is downregulated after operation. It showed an AUC of 0.790 for GC diagnosis, which is significantly better than that of CEA and CA 72–4, the current clinical GC-related tumor markers [59]. The expression of circSMARCA5 is significantly attenuated in GC tissues and cell lines, with an AUC of 0.806 for GC diagnosis [60]. Similarly, hsa_circ_0000745 is significantly downregulated in GC tissues and plasma. The AUC of plasma hsa_circ_0000745 was 0.683 for GC diagnosis, but it increased to 0.775 when combined with CEA [61]. Han et al. [62] showed that the diagnostic efficacies of hsa_circ_0021087 and hsa_circ_0005051, when used individually, were comparable to that of CEA. When combined with CEA, however, the AUC increased to 0.7988, suggesting that the combination of hsa_circ_0021087, hsa_circ_0005051, and CEA can serve as a noninvasive panel for GC diagnosis. In conclusion, circRNAs can be reliable markers for GC diagnosis, especially early diagnosis, and their diagnostic efficiency can be enhanced by combining them with commonly used GC-related tumor markers.

## CircRNAs play a role in the development of drug resistance in GC

Currently, the primary treatments for advanced GC include neoadjuvant chemotherapy, radiotherapy, immunotherapy, and molecular targeted therapy, either individually or in combination. Despite showing unsatisfactory therapeutic efficiency in most patients, chemotherapy remains a crucial antitumor strategy. However, some tumor cells possess primary drug resistance before being exposed to chemotherapeutic drugs, while others develop secondary or acquired resistance after drug exposure. The latter is one of the primary reasons for the low survival rate of GC patients. In addition to coding genes, noncoding RNAs, especially circRNAs, have recently been shown to mediate drug resistance in GC cells [63, 64]. In this part, we summarized circRNAs linked to drug resistance in GC (Table 1).

**Table 1:** CircRNAs involved in treatment resistant of GC

No	CircRNA	Drugs or Others	Expression	Role in drug resistance	Molecular mechanism	Refs.
1	hsa_circ_0081143	Cisplatin	Upregulated	Enhancer	Sponge miR-646 to upregulate CDK6	[65]
2	circDONSON	Cisplatin	Upregulated	Enhancer	Sponge miR-802 to upregulate BMI1	[66]
3	circAKT3	Cisplatin	Upregulated	Enhancer	Sponge miR-198 to upregulate PIK3R1	[67]
4	circPVT1	Cisplatin	Upregulated	Enhancer	Sponge miR-152-3p to upregulate HDGF	[68]
5	circARVCF	Cisplatin	Upregulated	Enhancer	Sponge miR-1205 to upregulate FGFR1	[69]
6	circ-LDLRAD3	Cisplatin	Upregulated	Enhancer	Sponge miR-588 to upregulate SOX5	[70]
7	circ_0017274	Cisplatin	Upregulated	Enhancer	Sponge miR-637 to upregulate CDX2	[71]
8	circUBAP2	Cisplatin	Downregulated	Suppressor	Sponge miR-300 to upregulate KAT6B	[72]
9	circVAPA	Cisplatin	Upregulated	Enhancer	Sponge miR-125b-5p to upregulate STAT3	[73]
10	circCCDC66	Cisplatin	Upregulated	Enhancer	Sponge miR-618 to upregulate BCL2	[74]
11	circFN1	Cisplatin	Upregulated	Enhancer	Sponge miR-182-5p	[75]
12	circ_0000260	Cisplatin	Upregulated	Enhancer	Sponge miR-129-5p to upregulate MMP11	[76]
13	circCUL2	Cisplatin	Downregulated	Suppressor	Sponge miR-142-3p to upregulate ROCK2	[77]
14	circMCTP2	Cisplatin	Downregulated	Suppressor	Sponge miR-99a-5p to upregulate MTMR3	[78]
15	circHIPK3	Cisplatin	Upregulated	Enhancer	Sponge miR-508-3p to upregulate BCL-2	[79]
16	circSOD2	Cisplatin	Upregulated	Enhancer	Sponge miR-1296 to upregulate STAT1	[80]
17	circ-MTO1	Oxaliplatin	Downregulated	Suppressor	-	[81]
18	circLRCH3	Oxaliplatin	Upregulated	Enhancer	Sponge miR-383-5p to upregulate FGF7	[82]
19	hsa_circ_0001546	Oxaliplatin	Downregulated	Suppressor	Sponge miR-421 to upregulate ATM	[83]
20	circ_0032821	Oxaliplatin	Upregulated	Enhancer	Sponge miR-515-5p to upregulate SOX9	[84]
21	hsa_circ_0000144	Oxaliplatin	Upregulated	Enhancer	Sponge miR-502-5p to upregulate ADAM9	[85]
22	circMTHFD2	Pemetrexed	Upregulated	Enhancer	Sponge miR-124 to upregulate MDR-1	[86]
23	circ-PVT1	Pemetrexed	Upregulated	Enhancer	Sponge miR-124-3p/ to upregulate ZEB1	[87]
24	circCPM	5-FU	Upregulated	Enhancer	Sponge miR-21-3p to upregulate PRKAA2	[88]
25	circNRIP1	5-FU	Upregulated	Enhancer	Sponge miR-138-5p to upregulate MDR-1	[89]
26	circPRRX1	radiation	Upregulated	Enhancer	Sponge miR-596 to upregulate NKAP	[90]
27	circDLG1	anti-PD-1	Upregulated	Enhancer	Sponge miR-141-3p to upregulate CXCL12	[91]
28	hsa_circ_0000520	Herceptin	Downregulated	Suppressor	Inhibiting PI3K-Akt signaling pathway	[92]

### CircRNAs associated with chemotherapeutic drug resistance in GC

CircRNAs have been shown to be involved in the resistance of GC cells to platinum-based drugs. Hsa_circ_0081143, which is significantly upregulated in GC tissues, “sponged” miR-646, leading to the upregulation of *CDK6* and the promotion of resistance to cis-diamino-dichloro-platinum (II) (CDDP) in SGC7901 and MGC803 cells [65]. CircDONSON, which is significantly upregulated in both GC tissues and cell lines, also promoted CDDP resistance in AGS and HGC-27 cells by upregulating BMI1 via miR-802 adsorption [66]. Moreover, circAKT3 (hsa_circ_0000199), constituted by exons 8–11 of *AKT3*, was found to be significantly overexpressed in CDDP-resistant SGC7901 and BGC823 cells. When circAKT3 was knocked down, the sensitivity of SGC7901 cells to CDDP significantly increased. Mechanistically, circAKT3 establishes CDDP resistance in GC cells by adsorbing miR-198, which indirectly promotes the expression of PI3K regulatory subunit 1 [67]. CircPVT1 was found to be markedly upregulated in chemoresistant GC tissues and CDDP-resistant cells, and its knockdown increased sensitivity to CDDP via a mechanism involving the hepatoma-derived growth factor/PI3K/AKT pathway and the “sponging” of miR-152-3p. It also decreased cell viability and proliferation while inducing apoptosis in CDDP-resistant GC cells [68]. CircARVCF was upregulated in CDDP-resistant GC tissues and cells, and knocking it down repressed CDDP-resistant GC via miR-1205 [69]. The knockdown of circ-LDLRAD3, which was overexpressed in CDDP-resistant GC tissues and cells, increased the sensitivity of tumor cells to CDDP and attenuated cell proliferation, survival, and invasion by adsorbing miR-588 [70]. In addition, other circRNAs, such as circ_0017274, circUBAP2, circVAPA, circRNACCDC66, circFN1, circ_0000260, circCUL2, circMCTP2, circHIPK3, and circSOD2, have also been implicated in mediating CDDP resistance in GC through their miRNA- “sponging” functions [71–80].

Oxaliplatin, a third-generation platinum anticancer drug, is extensively used to treat GC, but the development of oxaliplatin resistance is a formidable barrier to successful treatment. CircRNAs have been implicated in the development of oxaliplatin resistance. Circ-MTO1 is usually underexpressed in gastric tumor tissues compared with adjacent tissues, and its overexpression enhanced the sensitivity of GC cells to oxaliplatin [81]. Knockdown of circLRCH3, which was found upregulated in oxaliplatin-resistant GC tissues and cells, mitigated oxaliplatin resistance through the miR-383-5p/fibroblast growth factor 7 axis [82]. Hsa_circ_0001546, which is significantly downregulated in GC cells, was found to adsorb miRNA-421 to activate the ataxia-telangiectasia mutated/checkpoint kinase 2/p53 signaling pathway, eventually suppressing oxaliplatin resistance in HGC-27 cells [83]. Circ_0032821 was reported to be highly expressed in oxaliplatin-resistant GC cells and their exosomes. Circ_0032821 regulates the expression of SRY-box transcription factor 9 by “sponging” miR-515-5p, ultimately facilitating proliferation, metastasis, and oxaliplatin resistance in GC cells [84]. Hsa_circ_0000144 was also found to be highly expressed in oxaliplatin-resistant GC tissues and cells, wherein it promotes oxaliplatin resistance by “sponging” miR-502-5p [85].

Besides platinum-based chemotherapy drugs, circRNAs have been implicated in the resistance of GC cells to other antitumor drugs. CircMTHFD2 enhances pemetrexed resistance in GC cells by upregulating multidrug resistance protein-1 via miR-124 adsorption [86]. Circ-PVT1 was found to be upregulated in paclitaxel-resistant GC tissues and cells, and its downregulation enhanced paclitaxel sensitivity in resistant cells by negatively regulating the miR-124-3p/zinc finger E-box-binding homeobox 1 axis [87]. The expression of circCPM was reported to be significantly elevated in GC cells and tissues resistant to 5-fluorouracil (5-FU). CircCPM adsorbs miR-21-3p to upregulate autophagy mediated by protein kinase AMP-activated catalytic subunit alpha 2, which eventually promotes 5-FU resistance in GC cells [88]. Overexpression of circNRIP1 in GC cells attenuated their sensitivity to 5-FU by upregulating multidrug resistance protein-1, P-glycoprotein, and HIF-1α through interactions with miR-138-5p [89]. All these studies demonstrate the different mechanisms by which circRNAs contribute to the development of drug resistance in GC cells.

### CircRNAs associated with resistance to radiotherapy or targeted therapy in GC

Exosomal circPRRX1 was found to be upregulated in human GC and could be transferred between cells via exosomes. Mechanistically, exosomal circPRRX1 promoted radiation resistance in GC cells through competing endogenous RNA crosstalk via the circPRRX1/miR-596/nuclear factor-κB-activating protein axis [90]. Interestingly, circRNAs can also regulate the sensitivity of GC cells to targeted therapy, such as anti-programmed cell death protein-1 (anti-PD-1) therapy. CircDLG1 was found to be markedly upregulated in distant metastatic lesions and GC tissues resistant to anti-PD-1 therapy. CircDLG1 interactes with miR-141-3p to upregulate C-X-C motif chemokine ligand 12, ultimately inducing resistance to anti-PD-1 therapy [91]. Hsa_circ_0000520 is downregulated in GC tissues and cell lines, and its overexpression can reverse Herceptin resistance in GC cells by inhibiting the PI3K/Akt signaling pathway [92].

In conclusion, these studies uncovered the critical role of circRNAs in mediating the resistance of GC cells to chemotherapeutic drugs or other treatments commonly used in the clinical setting. Therefore, targeting circRNAs can serve as a new strategy to potentially reverse drug resistance in GC.

## CircRNAs can serve as prognostic biomarkers for GC

CircRNAs are not only implicated in the tumorigenesis of GC but also significantly associated with its prognosis. CircOSBPL10, derived from the second and third exons of *OSBPL10* pre-mRNA, is notably upregulated in GC tissues, and is associated with lower overall survival and disease-free survival rates [93]. Similarly, hsa_circ_0010882 is substantially upregulated in GC tissues and cell lines, and its expression is positively associated with tumor size and TNM stage. Therefore, a group of patients with higher expression of hsa_circ_0010882 exhibited lower overall survival rates [94]. Hsa_circ_0009910 is significantly upregulated in GC cells and is positively correlated with TNM stage, distant metastasis, and differentiation; therefore, patients with higher expression of hsa_circ_0009910 had worse prognoses [95]. Hsa_circ_0001020 is also significantly upregulated in GC cell lines, tissues, and patient sera and is negatively correlated with overall survival [96]. Therefore, circRNAs can serve as potential prognostic biomarkers in GC.

## Application of circRNAs as biotechnological drugs for treating GC

**Fig. 5:**
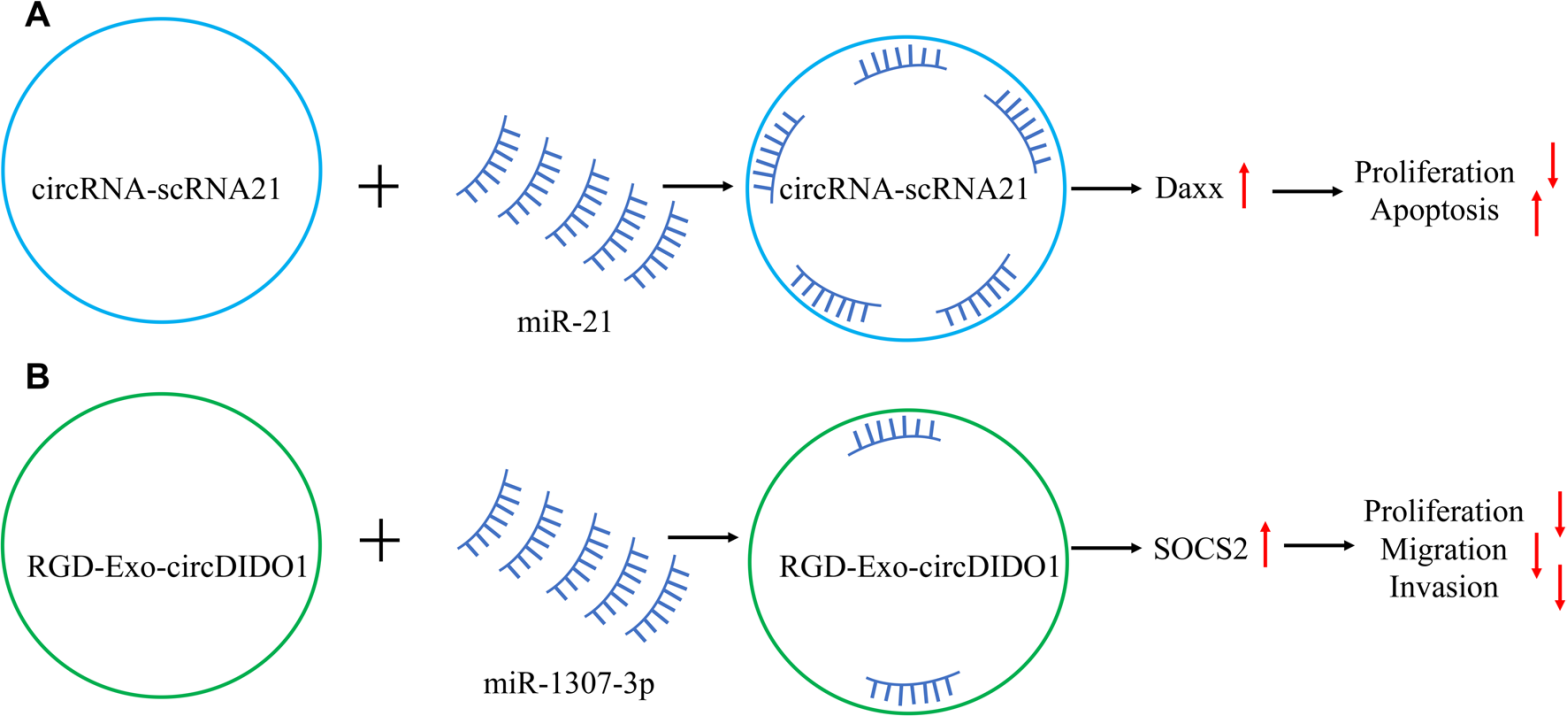
Application of circRNAs as biotechnological drugs for the treatment of GC. **A**. Artificially synthesized circRNA-scRNA21 acts as a sponge for miR-21, upregulating *Daxx* and suppressing cell proliferation while promoting the apoptosis of GC cells. **B**. CircDIDO1-loaded, RGD-modified exosomes inhibited the proliferation, migration, and invasion of GC cells through the miR-1307-3p/SOCS2 pathway

Nucleic acid drugs hold great promise for various applications. Substantial evidence exists for the essential roles played by circRNAs in the development, progression, diagnosis, and prognosis of GC. “Sponging” of miRNAs is one of the most important mechanisms employed by circRNAs, while other molecular mechanisms are currently being investigated. Therefore, circRNAs undoubtedly carry significant potential as anti-GC therapeutic agents. Liu et al. [97] synthetically created a circRNA, scRNA21, to investigate its ability to “sponge” miR-21. ScRNA21, which included five regions that could bind miR-21, was more resistant to nuclease degradation and was significantly more stable in vitro than its linear RNA counterparts. Functionally, scRNA21 could evidently inhibit GC cell proliferation and promoted apoptosis by sponging miR-21. Furthermore, scRNA21 could reverse the downregulation of miR-21-linked downstream target genes. Mechanistically, scRNA21 overexpression significantly suppressed the proliferation of GC cells by upregulating the tumor suppressor gene *Daxx* (Fig. 5A). Therefore, circRNAs can serve as nucleic acid drugs in the treatment of GC.

A research group has investigated the efficacy of RGD-modified exosomes loaded with circDIDO1 (RGD-Exo-circDIDO1) in GC therapy (Fig. 5B). RGD-Exo-circDIDO1 efficiently delivered circDIDO1 to GC cells while substantially suppressing their proliferation, migration, and invasion via the miR-1307-3p/suppressor of cytokine signaling 2 axis [98]. Surprisingly, RGD-Exo-circDIDO1 treatment did not elicit any inflammatory response or notable hepatic or renal toxicity, indicating that it could serve as a promising approach for GC therapy with minimal side effects [98]. These findings suggest that the development of circRNA-based biotechnological drugs for cancer treatment represents a potential approach to combat malignant tumors.

## Expression of circRNAs in GC can be regulated by drugs and microorganisms

Recent research has demonstrated that certain therapeutic agents used in clinical settings can impact the biological function of tumor cells by regulating circRNA expression (Table 2). Lidocaine, a widely used local anesthetic, was shown to significantly inhibit the proliferation and metastasis of GC cells. Lidocaine upregulates circ_ANO5, which can adsorb miR-21-5p and elevate the expression of leukemia inhibitory factor receptor [99]. Astragaloside IV (AS-IV), a monomer compound isolated from *Astragalus membranaceus* Bunge, can inhibit the proliferation, invasion, and metastasis of various types of cancer cells through different pathways [100–103]. Interestingly, AS-IV can also suppress cancer cell growth by modulating circRNA expression. For instance, AS-IV inhibited the proliferation, migration, and invasion of GC cells by downregulating circDLST, which attenuated the expression of eukaryotic translation initiation factor 4A1 through miR-489-3p adsorption [104]. Icariin, a natural compound found in *Epimedium* herbs, enhanced hsa_circ_0003159 expression and reduced GC cell proliferation by regulating the hsa_circ_0003159/miR-223-3p/NLR family pyrin domain-containing protein 3 signaling axis [105]. Sinomenine, an alkaloid extracted from the rhizome of the Chinese herb *Sinomenium acutum (Thunb.)Rehd.et Wils.* [106], downregulated circTRPM7 and inhibited the proliferation and metastasis of GC through the circTRPM7/miR-145-5p/pre-B cell leukemia homeobox 3 axis [107].

**Table 2:** CircRNAs regulated by drugs or microorganisms in GC

No	Drugs or Microorganisms	CircRNA	Expression	Property in GC tumorigenesis or progression	Molecular mechanism	Refs.
1	Lidocaine	circ_ANO5	Downregulated	Repressor	Sponge miR-21-5p to upregulate LIFR	[99]
2	Astragaloside IV	circDLST	Upregulated	Enhancer	Sponge miR-489-3p to upregulate EIF4A1	[104]
3	Icariin	hsa_circ_0003159	Downregulated	Repressor	Sponge miR-223-3p to upregulate NLRP3	[105]
4	Sinomenine	circTRPM7	Upregulated	Enhancer	sponge miR-145-5p to upregulate PBX3	[107]
5	Berberine	31 circRNAs	Upregulated (19), Downregulated (12)	–	–	[111]
6	H. pylori	circMAN1A2	Upregulated	Enhancer	Sponge miR-1236-3p to upregulate MTA2	[116]
7	H. pylori	circFNDC3B	Upregulated	Enhancer	Sponge miR-942 to upregulate CD44 and miR-510 to upregulate CDH1	[117]
8	H. pylori	circRNA_15430	Downregulated	Repressor	Sponge miR-382-5p to downregulate ZCCHC14	[118]

Instead of modulating the expression of individual circRNAs, some drugs can impact tumorigenesis or progression of GC by regulating the abundance of circRNAs. Berberine, a plant alkaloid extracted from traditional Chinese herbs [108], exerts ameliorative effects on blood glucose, blood lipids, and blood pressure [109, 110]. Recently, berberine was also shown to significantly inhibit GC cell proliferation and regulate the expression of 31 circRNAs, which subsequently modulate the Notch, mitogen-activated protein kinase, and nuclear factor-κB signaling pathways through competing endogenous RNA mechanisms [111].

Some microorganisms have also been shown to modulate circRNA expression. *H. pylori* is an important initial factor for the development of GC [112, 113], and it regulates the expression of some noncoding RNAs during GC tumorigenesis or progression [114–117]. For example, *H. pylori* upregulated circMAN1A2 in AGS and BGC823 cells independent of cytotoxin-associated gene A [116]. CircMAN1A2 “sponges” miR-1236-3p to regulate the expression of metastasis-associated protein 2, thereby promoting the carcinogenesis of *H. pylori*-induced GC [116]. CircFNDC3B aggravated the recurrence of *H. pylori*-induced early GC via the miR-942/CD44 and miR-510/CDH1 pathway [117]. On the other hand, *H. pylori* can also downregulate certain circRNAs. The expression of circRNA_15430 was reduced in *H. pylori*-positive gastritis tissues and *H. pylori*-infected MGC-803 cells, which promoted the proliferation and migration of GC cells while suppressing apoptosis and autophagy through the miR-382-5p/zinc finger CCHC-type containing 14 pathways [118]. These results demonstrate that circRNAs can be developed as therapeutic targets.

## Conclusions and future perspectives

CircRNAs have emerged as key players in the carcinogenesis and progression of GC, presenting promising prospects as potential diagnostic and prognostic biomarkers. The bulk of circRNA research in GC has thus far zoomed in on their role as microRNA “sponges”, their interactions with RBPs or other proteins, and their capacity to act as templates for translation. Beyond this, synthetic circRNAs have demonstrated notable anticancer activities via effective miRNA sequestration. Advancements in circRNA research have not only enhanced our understanding of GC etiology but also identified prospective targets for diagnosis and therapy.

Despite significant strides in the circRNA domain, the journey is far from complete, as hurdles persist. A primary obstacle is the enigmatic nature of circRNA biogenesis in humans. Recent studies suggest a correlation between circRNA biogenesis and specific RBPs in GC. However, the precise mechanisms underpinning circRNA dysregulation in GC largely remain unexplored. Moreover, the typically low expression levels of circRNAs in human tissue challenge detection, with real-time fluorescence quantitative PCR (qPCR) being the most prevalent yet not always adequate technique. Particularly, unsuccessful “divergent primers” designs have rendered some circRNAs undetectable by qPCR. Consequently, there’s a pressing need for more sensitive detection methodologies suitable for both cancer tissue and patient serum. Adding to this, the current body of circRNA research in GC has narrowly focused on functions through limited mechanisms, whereas novel roles attributable to their intricate secondary structures await discovery. Another formidable challenge involves in vitro expression, raising circRNA levels has proven difficult, ushering an urgent call for innovative approaches to elevate the in vitro expressing efficiency of circRNA for their functional study of GC.

Accounting for the variable expression of circular RNAs across different cell types, developmental stages, and species, we must tread cautiously when generalizing findings, be it from in vitro studies such as cell cultures to in vivo environments like animal models, or cross-species extrapolations. And while aberrant circRNA expression is documented in specific tumor types, establishing a direct and unequivocal link between circRNAs and diseases necessitates more concrete evidence. Accordingly, there is a pressing need for further research to decode their precise roles in disease progression. Furthermore, although strides have been made in decoding the action mechanisms of circRNAs in tumors, these insights are yet to translate into practical interventions or therapies.

Nonetheless, the advances in circRNA research in GC are clear. The unique covalently closed loop structure of circRNAs imbues them with greater stability than linear mRNAs in cells and bodily fluids, positioning them favorably for clinical application. These features of circRNAs highlight their potential as robust biomarkers and therapeutic targets, paving the way for innovative advances in the fight against GC.

## Data Availability

Not applicable.

## References

[1] Sung H, Ferlay J, Siegel RL, Laversanne M, Soerjomataram I, Jemal A (2021). Global cancer statistics 2020: GLOBOCAN estimates of incidence and mortality worldwide for 36 cancers in 185 countries. CA Cancer J Clin.

[2] Smyth EC, Nilsson M, Grabsch HI, van Grieken NC, Lordick F (2020). Gastric cancer. Lancet.

[3] Jgc A (2011). Japanese classification of gastric carcinoma. Gastric Cancer.

[4] Lauren P (1965). The two histological main types of gastric carcinoma: diffuse and so-called intestinal-type carcinoma. an attempt at a histo-clinical classification. Acta Pathol Microbiol Scand.

[5] WHO (2019). Digestive system tumours WHO classification of tumours.

[6] Karimi P, Islami F, Anandasabapathy S, Freedman ND, Kamangar F (2014). Gastric cancer: descriptive epidemiology, risk factors, screening, and prevention. Cancer Epidemiol Biomarkers Prev.

[7] Varambally S, Cao Q, Mani RS, Shankar S, Wang X, Ateeq B (2008). Genomic loss of microRNA-101 leads to overexpression of histone methyltransferase EZH2 in cancer. Science.

[8] Cai Z, Shi Q, Li Y, Jin L, Li S, Wong L (2023). LncRNA EILA promotes CDK4/6 inhibitor resistance in breast cancer by stabilizing cyclin E1 protein. Sci Adv..

[9] Ruan Y, Chen T, Zheng L, Cai J, Zhao H, Wang Y (2023). cDCBLD2 mediates sorafenib resistance in hepatocellular carcinoma by sponging miR-345-5p binding to the TOP2A coding sequence. Int J Biol Sci.

[10] Jeck WR, Sorrentino JA, Wang K, Slevin MK, Burd CE, Liu J (2013). Circular RNAs are abundant, conserved, and associated with ALU repeats. RNA.

[11] Kolakofsky D (1976). Isolation and characterization of Sendai virus DI-RNAs. Cell.

[12] Hsu MT, Coca-Prados M (1979). Electron microscopic evidence for the circular form of RNA in the cytoplasm of eukaryotic cells. Nature.

[13] Kelly S, Greenman C, Cook PR, Papantonis A (2015). Exon skipping is correlated with exon circularization. J Mol Biol.

[14] Kristensen LS, Andersen MS, Stagsted LVW, Ebbesen KK, Hansen TB, Kjems J (2019). The biogenesis, biology and characterization of circular RNAs. Nat Rev Genet.

[15] Schmidt CA, Giusto JD, Bao A, Hopper AK, Matera AG (2019). Molecular determinants of metazoan tricRNA biogenesis. Nucleic Acids Res.

[16] Huang G, Li S, Yang N, Zou Y, Zheng D, Xiao T (2017). Recent progress in circular RNAs in human cancers. Cancer Lett.

[17] Xiong H, Huang G, Zhu Y, Chen R, Zuo L, Liu H (2023). Circ-SHPRH in human cancers: a systematic review and meta-analysis. Front Cell Dev Biol.

[18] Sun K, Yao H, Zhang P, Sun Y, Ma J, Xia Q (2023). Emerging landscape of circFNDC3B and its role in human malignancies. Front Oncol.

[19] Khalilian S, Mohajer Z, Khazeei Tabari MA, Ghobadinezhad F, Ghafouri-Fard S (2023). circGFRA1: a circular RNA with important roles in human carcinogenesis. Pathol Res Pract.

[20] Wang X, Zhang C, Song H, Yuan J, Zhang L, He J (2022). CircCCDC66: Emerging roles and potential clinical values in malignant tumors. Front Oncol.

[21] Wu X, Shi M, Lian Y, Zhang H (2023). Exosomal circRNAs as promising liquid biopsy biomarkers for glioma. Front Immunol.

[22] Guo X, Gao C, Yang D, Li S (2023). Exosomal circular RNAs: a chief culprit in cancer chemotherapy resistance. Drug Resist Updat.

[23] Wang Z, Ma K, Pitts S, Cheng Y, Liu X, Ke X (2019). Novel circular RNA circNF1 acts as a molecular sponge, promoting gastric cancer by absorbing miR-16. Endocr Relat Cancer.

[24] Bu X, Zhang X, Luan W, Zhang R, Zhang Y, Zhang A (2020). Next-generation sequencing reveals hsa_circ_0058092 being a potential oncogene candidate involved in gastric cancer. Gene.

[25] Cai X, Nie J, Chen L, Yu F (2020). Circ_0000267 promotes gastric cancer progression via sponging MiR-503-5p and regulating HMGA2 expression. Mol Genet Genomic Med.

[26] Li C, Peng X, Peng Z, Yan B (2022). circBGN accelerates gastric cancer cell proliferation and invasion via activating IL6/STAT3 signaling pathway. FASEB J.

[27] Fan H, Chen Z, Chen X, Chen M, Yi Y, Zhu J (2022). METTL14-mediated m(6)A modification of circORC5 suppresses gastric cancer progression by regulating miR-30c-2-3p/AKT1S1 axis. Mol Cancer.

[28] Liang X, Qin C, Yu G, Guo X, Cheng A, Zhang H (2021). Circular RNA circRAB31 acts as a miR-885-5psponge to suppress gastric cancer progressionvia the PTEN/PI3K/AKT pathway. Mol Ther Oncolytics.

[29] Xu P, Zhang X, Cao J, Yang J, Chen Z, Wang W (2022). The novel role of circular RNA ST3GAL6 on blocking gastric cancer malignant behaviours through autophagy regulated by the FOXP2/MET/mTOR axis. Clin Transl Med.

[30] Hong Y, Qin H, Li Y, Zhang Y, Zhuang X, Liu L (2019). FNDC3B circular RNA promotes the migration and invasion of gastric cancer cells via the regulation of E-cadherin and CD44 expression. J Cell Physiol.

[31] Ma Q, Yang F, Huang B, Pan X, Li W, Yu T (2022). CircARID1A binds to IGF2BP3 in gastric cancer and promotes cancer proliferation by forming a circARID1A-IGF2BP3-SLC7A5 RNA-protein ternary complex. J Exp Clin Cancer Res.

[32] Chen Y, Yang F, Fang E, Xiao W, Mei H, Li H (2019). Circular RNA circAGO2 drives cancer progression through facilitating HuR-repressed functions of AGO2-miRNA complexes. Cell Death Differ.

[33] Yang H, Yuan W, Shang W, Wang H, Ning S, Liu J (2023). circSLC4A7 accelerates stemness and progression of gastric cancer by interacting with HSP90 to activate NOTCH1 signaling pathway. Cell Death Dis.

[34] Yang F, Ma Q, Huang B, Wang X, Pan X, Yu T (2023). CircNFATC3 promotes the proliferation of gastric cancer through binding to IGF2BP3 and restricting its ubiquitination to enhance CCND1 mRNA stability. J Transl Med.

[35] Dong J, Zheng Z, Zhou M, Wang Y, Chen J, Cen J (2023). EGCG-LYS Fibrils-mediated CircMAP2K2 silencing decreases the proliferation and metastasis ability of gastric cancer cells in vitro and in vivo. Adv Sci.

[36] Yang F, Hu A, Li D, Wang J, Guo Y, Liu Y (2019). Circ-HuR suppresses HuR expression and gastric cancer progression by inhibiting CNBP transactivation. Mol Cancer.

[37] Yuan Y, Zhang X, Du K, Zhu X, Chang S, Chen Y (2022). Circ_CEA promotes the interaction between the p53 and cyclin-dependent kinases 1 as a scaffold to inhibit the apoptosis of gastric cancer. Cell Death Dis.

[38] Qiu S, Li B, Xia Y, Xuan Z, Li Z, Xie L (2022). CircTHBS1 drives gastric cancer progression by increasing INHBA mRNA expression and stability in a ceRNA- and RBP-dependent manner. Cell Death Dis.

[39] Zang X, Jiang J, Gu J, Chen Y, Wang M, Zhang Y (2022). Circular RNA EIF4G3 suppresses gastric cancer progression through inhibition of β-catenin by promoting δ-catenin ubiquitin degradation and upregulating SIK1. Mol Cancer.

[40] Du Y, Zhang J, Gong L, Feng Z, Wang D, Pan Y (2022). Hypoxia-induced ebv-circLMP2A promotes angiogenesis in EBV-associated gastric carcinoma through the KHSRP/VHL/HIF1α/VEGFA pathway. Cancer Lett.

[41] Geng X, Wang J, Zhang C, Zhou X, Jing J, Pan W (2021). Circular RNA circCOL6A3_030 is involved in the metastasis of gastric cancer by encoding polypeptide. Bioengineered.

[42] Peng Y, Xu Y, Zhang X, Deng S, Yuan Y, Luo X (2021). A novel protein AXIN1-295aa encoded by circAXIN1 activates the Wnt/β-catenin signaling pathway to promote gastric cancer progression. Mol Cancer.

[43] Hu F, Peng Y, Chang S, Luo X, Yuan Y, Zhu X (2022). Vimentin binds to a novel tumor suppressor protein, GSPT1-238aa, encoded by circGSPT1 with a selective encoding priority to halt autophagy in gastric carcinoma. Cancer Lett.

[44] Jiang T, Xia Y, Lv J, Li B, Li Y, Wang S (2021). A novel protein encoded by circMAPK1 inhibits progression of gastric cancer by suppressing activation of MAPK signaling. Mol Cancer.

[45] Zhang Y, Jiang J, Zhang J, Shen H, Wang M, Guo Z (2021). CircDIDO1 inhibits gastric cancer progression by encoding a novel DIDO1-529aa protein and regulating PRDX2 protein stability. Mol Cancer.

[46] Liu H, Fang D, Zhang C, Zhao Z, Liu Y, Zhao S (2023). Circular MTHFD2L RNA-encoded CM-248aa inhibits gastric cancer progression by targeting the SET-PP2A interaction. Mol Ther.

[47] Wang X, Li J, Bian X, Wu C, Hua J, Chang S (2021). CircURI1 interacts with hnRNPM to inhibit metastasis by modulating alternative splicing in gastric cancer. Proc Natl Acad Sci USA.

[48] Jie M, Wu Y, Gao M, Li X, Liu C, Ouyang Q (2020). CircMRPS35 suppresses gastric cancer progression via recruiting KAT7 to govern histone modification. Mol Cancer.

[49] Shao Y, Tao X, Lu R, Zhang H, Ge J, Xiao B (2020). Hsa_circ_0065149 is an indicator for early gastric cancer screening and prognosis prediction. Pathol Oncol Res.

[50] Lu H, Zhang Z, Yuan X, Song H, Li P (2023). The role of circular RNA hsa_circ_0001789 as a diagnostic biomarker in gastric carcinoma. Scand J Gastroenterol.

[51] Shao Y, Qi C, Yan J, Lu R, Ye G, Guo J (2022). Biological and clinical implications of hsa_circ_0086720 in gastric cancer and its clinical application. J Clin Lab Anal.

[52] Wang Y, Xu S, Chen Y, Zheng X, Li T, Guo J (2019). Identification of hsa_circ_0005654 as a new early biomarker of gastric cancer. Cancer Biomark.

[53] Ma S, Yao Y, Xu Y, Zou M, Zhou M, Abudushalamu G (2024). Comprehensive evaluation of serum circHAS2 as a novel diagnostic and prognostic biomarker for gastric cancer. Mol Carcinog.

[54] Yang X, Xia J, Peng C, Cai W (2023). Expression of plasma exosomal circLPAR1 in patients with gastric cancer and its clinical application value. Am J Cancer Res.

[55] Li X, Lin Y, Shao J, Wu X, Li X, Yao H (2023). Plasma exosomal hsa_circ_0079439 as a novel biomarker for early detection of gastric cancer. World J Gastroenterol.

[56] Zheng P, Gao H, Xie X, Lu P (2022). Plasma exosomal hsa_circ_0015286 as a potential diagnostic and prognostic biomarker for gastric cancer. Pathol Oncol Res.

[57] Lu J, Zhang P, Xie J, Wang J, Lin J, Chen Q (2019). Circular RNA hsa_circ_0006848 related to ribosomal protein L6 acts as a novel biomarker for early gastric cancer. Dis Markers.

[58] Roy S, Kanda M, Nomura S, Zhu Z, Toiyama Y, Taketomi A (2022). Diagnostic efficacy of circular RNAs as noninvasive, liquid biopsy biomarkers for early detection of gastric cancer. Mol Cancer.

[59] Lu J, Zhang P, Xie J, Wang J, Lin J, Chen Q (2019). Hsa_circ_0000467 promotes cancer progression and serves as a diagnostic and prognostic biomarker for gastric cancer. J Clin Lab Anal.

[60] Cai J, Chen Z, Zuo X (2019). circSMARCA5 functions as a diagnostic and prognostic biomarker for gastric cancer. Dis Markers.

[61] Huang M, He Y, Liang L, Huang Q, Zhu Z (2017). Circular RNA hsa_circ_0000745 may serve as a diagnostic marker for gastric cancer. World J Gastroenterol.

[62] Han L, Zhang X, Wang A, Ji Y, Cao X, Qin Q (2020). A dual-circular rna signature as a non-invasive diagnostic biomarker for gastric cancer. Front Oncol.

[63] Marin JJG, Perez-Silva L, Macias RIR, Asensio M, Peleteiro-Vigil A, Sanchez-Martin A (2020). Molecular bases of mechanisms accounting for drug resistance in gastric adenocarcinoma. Cancers.

[64] Wei L, Sun J, Zhang N, Zheng Y, Wang X, Lv L (2020). Noncoding RNAs in gastric cancer: implications for drug resistance. Mol Cancer.

[65] Xue M, Li G, Fang X, Wang L, Jin Y, Zhou Q (2019). hsa_circ_0081143 promotes cisplatin resistance in gastric cancer by targeting miR-646/CDK6 pathway. Cancer Cell Int.

[66] Liu Y, Xu J, Jiang M, Ni L, Ling Y (2020). CircRNA DONSON contributes to cisplatin resistance in gastric cancer cells by regulating miR-802/BMI1 axis. Cancer Cell Int.

[67] Huang X, Li Z, Zhang Q, Wang W, Li B, Wang L (2019). Circular RNA AKT3 upregulates PIK3R1 to enhance cisplatin resistance in gastric cancer via miR-198 suppression. Mol Cancer.

[68] Wang X, Zhang Y, Li W, Liu X (2021). Knockdown of cir_RNA PVT1 elevates gastric cancer cisplatin sensitivity via sponging miR-152-3p. J Surg Res.

[69] Zhang R, Zhao H, Yuan H, Wu J, Liu H, Sun S (2021). CircARVCF contributes to cisplatin resistance in gastric cancer by altering miR-1205 and FGFR1. Front Genet.

[70] Liang Q, Chu F, Zhang L, Jiang Y, Li L, Wu H (2023). circ-LDLRAD3 knockdown reduces cisplatin chemoresistance and inhibits the development of gastric cancer with cisplatin resistance through miR-588 enrichment-mediated SOX5 inhibition. Gut Liver.

[71] Xu B, Guo J, Chen M (2022). Circ_0017274 acts on miR-637/CDX2 axis to facilitate cisplatin resistance in gastric cancer. Clin Exp Pharmacol Physiol.

[72] Cheng W, Luan P, Jin X (2023). circUBAP2 inhibits cisplatin resistance in gastric cancer via miR-300/KAT6B axis. Anticancer Drugs.

[73] Deng P, Sun M, Zhao W, Hou B, Li K, Zhang T (2021). Circular RNA circVAPA promotes chemotherapy drug resistance in gastric cancer progression by regulating miR-125b-5p/STAT3 axis. World J Gastroenterol.

[74] Zhang Q, Miao Y, Fu Q, Hu H, Chen H, Zeng A (2020). CircRNACCDC66 regulates cisplatin resistance in gastric cancer via the miR-618/BCL2 axis. Biochem Biophys Res Commun.

[75] Huang X, Zhang Q, Hu H, Jin Y, Zeng A, Xia Y (2020). A novel circular RNA circFN1 enhances cisplatin resistance in gastric cancer via sponging miR-182–5p. J Cell Biochem.

[76] Liu S, Wu M, Peng M (2020). Circ_0000260 regulates the development and deterioration of gastric adenocarcinoma with cisplatin resistance by upregulating MMP11 via targeting MiR-129-5p. Cancer Manag Res.

[77] Peng L, Sang H, Wei S, Li Y, Jin D, Zhu X (2020). circCUL2 regulates gastric cancer malignant transformation and cisplatin resistance by modulating autophagy activation via miR-142-3p/ROCK2. Mol Cancer.

[78] Sun G, Li Z, He Z, Wang W, Wang S, Zhang X (2020). Circular RNA MCTP2 inhibits cisplatin resistance in gastric cancer by miR-99a-5p-mediated induction of MTMR3 expression. J Exp Clin Cancer Res.

[79] Shang Z, Luo Z, Wang Y, Liu Q, Xin Y, Zhang M (2023). CircHIPK3 contributes to cisplatin resistance in gastric cancer by blocking autophagy-dependent ferroptosis. J Cell Physiol.

[80] Qu B, Liu J, Peng Z, Xiao Z, Li S, Wu J (2023). CircSOD2 polarizes macrophages towards the M1 phenotype to alleviate cisplatin resistance in gastric cancer cells by targeting the miR-1296/STAT1 axis. Gene.

[81] Chang C, Zheng A, Wang P, Teng X (2022). Circular RNA mitochondrial translation optimization 1 correlates with less lymph node metastasis, longer disease-free survival, and higher chemotherapy sensitivity in gastric cancer. J Clin Lab Anal.

[82] Xiang C, Li R, Qiu H, Zuo E, Zhang Y, Shan L (2023). Circular RNA circLRCH3 promotes oxaliplatin resistance in gastric cancer through the modulation of the miR-383-5p/FGF7 axis. Histol Histopathol.

[83] Wu Q, Wang H, Liu L, Zhu K, Yu W, Guo J (2020). Hsa_circ_0001546 acts as a miRNA-421 sponge to inhibit the chemoresistance of gastric cancer cells via ATM/Chk2/p53-dependent pathway. Biochem Biophys Res Commun.

[84] Zhong Y, Wang D, Ding Y, Tian G, Jiang B (2021). Circular RNA circ_0032821 contributes to oxaliplatin (OXA) resistance of gastric cancer cells by regulating SOX9 via miR-515-5p. Biotechnol Lett.

[85] Gao H, Xu J, Qiao F, Xue L (2021). Depletion of hsa_circ_0000144 suppresses oxaliplatin resistance of gastric cancer cells by regulating miR-502-5p/ADAM9 Axis. Onco Targets Ther.

[86] Xu Q, Xie M, Huang J, Wang Z (2019). Effect of circ MTHFD2 on resistance to pemetrexed in gastric cancer through regulating expression of miR-124. Eur Rev Med Pharmacol Sci.

[87] Liu Y, Zhang L, Du W (2019). Circular RNA circ-PVT1 contributes to paclitaxel resistance of gastric cancer cells through the regulation of ZEB1 expression by sponging miR-124-3p. Biosci Rep.

[88] Fang L, Lv J, Xuan Z, Li B, Li Z, He Z (2022). Circular CPM promotes chemoresistance of gastric cancer via activating PRKAA2-mediated autophagy. Clin Transl Med.

[89] Xu G, Li M, Wu J, Qin C, Tao Y, He H (2020). Circular RNA circNRIP1 sponges microRNA-138-5p to maintain hypoxia-induced resistance to 5-fluorouracil through HIF-1α-dependent glucose metabolism in gastric carcinoma. Cancer Manag Res.

[90] He Y, Zheng L, Yuan M, Fan J, Rong L, Zhan T (2022). Exosomal circPRRX1 functions as a ceRNA for miR-596 to promote the proliferation, migration, invasion, and reduce radiation sensitivity of gastric cancer cells via the upregulation of NF-κB activating protein. Anticancer Drugs.

[91] Chen D, Sheng H, Zhang D, Jin Y, Zhao B, Chen N (2021). The circular RNA circDLG1 promotes gastric cancer progression and anti-PD-1 resistance through the regulation of CXCL12 by sponging miR-141-3p. Mol Cancer.

[92] Lv X, Li P, Wang J, Gao H, Hei Y, Zhang J (2020). hsa_circ_0000520 influences herceptin resistance in gastric cancer cells through PI3K-Akt signaling pathway. J Clin Lab Anal.

[93] Wang S, Zhang X, Li Z, Wang W, Li B, Huang X (2019). Circular RNA profile identifies circOSBPL10 as an oncogenic factor and prognostic marker in gastric cancer. Oncogene.

[94] Peng Y, Pu K, Su H, Zhang J, Zheng Y, Ji R (2020). Circular RNA hsa_circ_0010882 promotes the progression of gastric cancer via regulation of the PI3K/Akt/mTOR signaling pathway. Eur Rev Med Pharmacol Sci.

[95] Liu M, Liu K, Zhang L, Cai J, Yao H, Bai Y (2018). Circ_0009910 regulates growth and metastasis and is associated with poor prognosis in gastric cancer. Eur Rev Med Pharmacol Sci.

[96] Yan J, Shao Y, Lu H, Ye Q, Ye G, Guo J (2022). Hsa_circ_0001020 serves as a potential biomarker for gastric cancer screening and prognosis. Dig Dis Sci.

[97] Liu X, Abraham JM, Cheng Y, Wang Z, Wang Z, Zhang G (2018). Synthetic circular RNA functions as a miR-21 sponge to suppress gastric carcinoma cell proliferation. Mol Ther Nucleic Acids.

[98] Guo Z, Zhang Y, Xu W, Zhang X, Jiang J (2022). Engineered exosome-mediated delivery of circDIDO1 inhibits gastric cancer progression via regulation of MiR-1307-3p/SOCS2 Axis. J Transl Med.

[99] Guan E, Liu H, Xu N (2022). Lidocaine suppresses gastric cancer development through Circ_ANO5/miR-21-5p/LIFR Axis. Dig Dis Sci.

[100] Min L, Wang H, Qi H (2022). Astragaloside IV inhibits the progression of liver cancer by modulating macrophage polarization through the TLR4/NF-κB/STAT3 signaling pathway. Am J Transl Res.

[101] Liu W, Chen H, Wang D (2021). Protective role of astragaloside IV in gastric cancer through regulation of microRNA-195-5p-mediated PD-L1. Immunopharmacol Immunotoxicol.

[102] Zhang C, Li L, Hou S, Shi Z, Xu W, Wang Q (2021). Astragaloside IV inhibits hepatocellular carcinoma by continually suppressing the development of fibrosis and regulating pSmad3C/3L and Nrf2/HO-1 pathways. J Ethnopharmacol.

[103] Wang X, Gao S, Song L, Liu M, Sun Z, Liu J (2021). Astragaloside IV antagonizes M2 phenotype macrophage polarization-evoked ovarian cancer cell malignant progression by suppressing the HMGB1-TLR4 axis. Mol Immunol.

[104] Li F, Cao K, Wang M, Liu Y, Zhang Y (2022). Astragaloside IV exhibits anti-tumor function in gastric cancer via targeting circRNA dihydrolipoamide S-succinyltransferase (circDLST)/miR-489-3p/ eukaryotic translation initiation factor 4A1(EIF4A1) pathway. Bioengineered.

[105] Zhang F, Yin Y, Xu W, Song Y, Zhou Z, Sun X (2022). Icariin inhibits gastric cancer cell growth by regulating the hsa_circ_0003159/miR-223-3p/NLRP3 signaling axis. Hum Exp Toxicol.

[106] He L, Li X, Zeng X, Duan H, Wang S, Lei L (2014). Sinomenine induces apoptosis in RAW 264.7 cell-derived osteoclasts in vitro via caspase-3 activation. Acta Pharmacol Sin..

[107] Yan J, Yang J, Shen H, Gao R, Lv S (2023). Sinomenine regulates circTRPM7-related pathway to inhibit gastric cancer cell growth and metastasis. Chem Biol Drug Des.

[108] Kumar A, Ekavali CK, Mukherjee M, Pottabathini R, Dhull DK (2015). Current knowledge and pharmacological profile of berberine: an update. Eur J Pharmacol.

[109] Derosa G, Maffioli P, Cicero AF (2012). Berberine on metabolic and cardiovascular risk factors: an analysis from preclinical evidences to clinical trials. Expert Opin Biol Ther.

[110] Tabeshpour J, Imenshahidi M, Hosseinzadeh H (2017). A review of the effects of Berberis vulgaris and its major component, berberine, in metabolic syndrome. Iran J Basic Med Sci.

[111] Wang M, Sun L, Wang L, Sun Y (2021). Effects of berberine on circular RNA expression profiles in human gastric cancer cells. Evid Based Complement Alternat Med.

[112] Guggenheim DE, Shah MA (2013). Gastric cancer epidemiology and risk factors. J Surg Oncol.

[113] Rieder G, Hofmann JA, Hatz RA, Stolte M, Enders GA (2003). Up-regulation of inducible nitric oxide synthase in Helicobacter pylori-associated gastritis may represent an increased risk factor to develop gastric carcinoma of the intestinal type. Int J Med Microbiol.

[114] Rao X, Liu X, Liu N, Zhang Y, Zhang Z, Zhou L (2021). Long noncoding RNA NEAT1 promotes tumorigenesis in H pylori gastric cancer by sponging miR-30a to regulate COX-2/BCL9 pathway. Helicobacter.

[115] Xin Z, Zhang L, Liu M, Wang Y, Zhang Y, Zhao W (2021). Helicobacter pylori infection-related long non-coding rna signatures predict the prognostic status for gastric cancer patients. Front Oncol.

[116] Guo R, Cui X, Li X, Zang W, Chang M, Sun Z (2022). CircMAN1A2 is upregulated by Helicobacter pylori and promotes development of gastric cancer. Cell Death Dis.

[117] Zhang J, Bai J, Zhu H, Li W, An Q, Wang D (2022). The upregulation of circFNDC3B aggravates the recurrence after endoscopic submucosal dissection (ESD) in early gastric cancer (EGC) patients. Sci Rep.

[118] Liu Y, Cao J, Yang Q, Zhu L, Zhao W, Wang X (2023). CircRNA_15430 reduced by Helicobacter pylori infection and suppressed gastric cancer progression via miR-382-5p/ZCCHC14 axis. Biol Direct.

